# Luteolin Suppresses Inflammatory Mediator Expression by Blocking the Akt/NF****κ****B Pathway in Acute Lung Injury Induced by Lipopolysaccharide in Mice

**DOI:** 10.1155/2012/383608

**Published:** 2011-11-20

**Authors:** Yi-Ching Li, Chung-Hsin Yeh, Ming-Ling Yang, Yu-Hsiang Kuan

**Affiliations:** ^1^Department of Pharmacology, School of Medicine, Chung Shan Medical University, No. 110, Sec.1, Jianguo N. Road, Taichung 40201, Taiwan; ^2^Department of Pharmacy, Chung Shan Medical University Hospital, Taichung, Taiwan; ^3^Department of Neurology, Show Chwan Memorial Hospital, Changhua, Taiwan; ^4^Graduate Institute of Life Sciences, National Chung Hsing University, Taichung, Taiwan; ^5^Department of Anatomy, School of Medicine, Chung Shan Medical University, No. 110, Sec.1, Jianguo N. Road, Taichung 40201, Taiwan

## Abstract

Acute lung injury (ALI), instilled by lipopolysaccharide (LPS), is a severe illness with excessive mortality and has no specific treatment strategy. Luteolin is an anti-inflammatory flavonoid and widely distributed in the plants. Pretreatment with luteolin inhibited LPS-induced histological changes of ALI and lung tissue edema. In addition, LPS-induced inflammatory responses, including increased vascular permeability, tumor necrosis factor (TNF)-**α** and interleukin (IL)-6 production, and expression of inducible nitric oxide synthase (iNOS) and cyclooxygenase-2 (COX-2), were also reduced by luteolin in a concentration-dependent manner. Furthermore, luteolin suppressed activation of NF**κ**B and its upstream molecular factor, Akt. These results suggest that the protection mechanism of luteolin is by inhibition of NF**κ**B activation possibly via Akt.

## 1. Introduction

Lipopolysaccharide (LPS) as an endotoxin is the major component of the outer wall membrane of Gram-negative bacteria and exists in high concentration in tobacco and tobacco smoke [1]. LPS is a potent trigger of septic shock and respiratory distress syndromes such as acute lung injury (ALI) and its most severe presentation, acute respiratory distress syndrome. However, ALI is not only strongly associated with mortality in critically ill patients but also linked with a high morbidity of approximately 34–58%. So far, no specific and effective treatment strategy for ALI has been established [2].

The physiologic characterizations of ALI are that increase in alveolar-capillary permeability, plasma protein leakage, protein-rich hyaline membrane formation, leukocytes infiltration, pulmonary edema, and haemorrhaging [3, 4]. Proinflammatory mediators such as tumor necrosis factor (TNF)-*α*, interleukin (IL)-6, nitric oxide (NO), and prostaglandin E2 (PGE2) play a critical role in the process of disease development in ALI [3]; while NO and PGE2 are generated by inducible nitric oxide synthase (iNOS) and cyclooxygenase (COX)-2, respectively, expression of TNF-*α*, IL-6, iNOS, and COX-2 are regulated by transcription factor, nuclear factor (NF)-*κ*B, activation [5]. In addition, protein kinase B (Akt/PKB), a serine/threonine kinase and a major downstream factor of phosphoinositide 3-kinase (PI3 K), modulates NF*κ*B activation in LPS-induced ALI [6].

Luteolin, a flavonoid, is widely distributed in vegetables, fruits, and herbs. In traditional Chinese medicine, plants rich in luteolin have been prescribed to treat hypertension, inflammatory diseases, and cancer. Luteolin has been reported of expressing anti-inflammatory, antioxidant, antiallergic, and antitumorigenic activities [7]. In macrophages, luteolin effectively inhibits LPS-induced proinflammatory cytokine expression and nitric oxide production [8]. Moreover, luteolin significantly reduces PMN activation, involving superoxide anion generation, leukotriene B4 secretion, migration, and chemotaxis [9, 10]. Recent study has shown that luteolin acts against PMN activation via the mitogen-activated protein kinase kinase/extracellular signal-regulated kinase (MEK/ERK) and PI3K/Akt pathways [11]. The aim of this study is to determine how luteolin exerts its anti-inflammatory function in ALI after LPS administration in *in vivo* animal model and the mechanism involved.

## 2. Materials and Methods

### 2.1. Materials

Mouse polyclonal antibodies to glyceraldehyde-3-phosphate dehydrogenase (GAPDH) Akt, iNOS, and COX-2 antibodies were obtained from Santa Cruz Biotechnology. Rabbit polyclonal antibodies to phospho-Akt were purchased from Cell Signaling Technology. Secondary antibodies were obtained from Jackson ImmunoResearch. Other chemicals were purchased from Sigma-Aldrich. The final volume of DMSO in the reaction mixture was <0.5%.

### 2.2. Animals

Adult healthy male ICR mice were purchased from BioLASCO Taiwan (Taipei, Taiwan), weighing 25–30 g. The mice were maintained on a standard diet with tap water *ad libidum* and housed in a 12 h light/dark cycle in a temperature-controlled environment (21 ± 1°C). The animals were cared for in accordance with guidelines provided by the Institutional Animal Ethics Committee of Chung Shan Medical University and conducted in accordance with the principles and guideline of the US National Institute of Health Guide for the Care and Use of Laboratory Animals.

### 2.3. Murine Model of LPS-Induced Lung Inflammation

LPS-induced ALI was performed as described in a previouse study [11]. Seventy-five mice were randomly divided into 5 groups: sham operation group and four treatment groups. The mice of the 4 treatment groups were injected with 0, 18, 35, or 70 *μ*mol/kg of luteolin intraperitoneally (I.P.) for 30 min, respectively, followed by intratracheal (I.T.) instillation of LPS (100 *μ*g/50 *μ*L); the sham operation group instead received vehicle I.P. for 30 min then followed by 50 *μ*L of saline I.T. After 6 hours, the mice were sacrificed by sodium pentobarbital, and samples were collected. In each group, the right lung was collected from 5 animals for histopathological studies, and the left lung for western blot and NF*κ*B activation assay. For the other 5 animals in each group, Bronchoalveolar lavage fluid (BALF) was collected for TNF-*α*, IL-6, and protein concentration assay, and lung samples for wet/dry weight ratio assay.

### 2.4. Lung Histopathological Studies

After midsternal thoracotomy, the mice underwent rapid excision of the lungs which were fixed via the trachea cannula with 4% isotonic paraformaldehyde. The end of trachea was tie off, and the whole lung was fixed additionally in paraformaldehyde for 24 h then embedded in paraffin after dehydration in a graded ethanol series and xylene. Serial sections of 3 *μ*m in thickness were performed using a rotatory microtome, and the deparaffinized sections were stained with hematoxylin and eosin. Evaluation of lung injury was performed under the light microscope.

### 2.5. Lung Wet/Dry Weight Ratio

The ratios are representative of lung edema. The left lung was obtained and the wet weight was recorded. The dry weight was determined after the lung was baked in an incubator at 80°C for 24 hours and the wet/dry weight ratios were then calculated.

### 2.6. Protein Concentration in BALF

Bronchoalveolar lavage was performed as previously described [11]. In short, after euthanasia, trachea was exposed and intubated with a tracheal cannula. Bronchoalveolar lavage was performed by flushing the airways and lungs repeatedly with 1 mL cold saline for three times. The pooled BALF was collected on ice and centrifuged at 500 *g* for 5 min at 4°C. Afterwards, the supernatant was stored at −20°C for further assay. Protein concentrations in the cell-free BALF were determined using Bio-Rad protein assay reagents. A standard curve was generated in the same fashion using bovine serum albumin.

### 2.7. TNF-*α* and IL-6 Analysis

The levels of TNF-*α* and IL-6 were measured on BALF using a commercially available ELISA kit (R & D Systems, Minneapolis, Minn, USA). These concentrations were interpolated from the standard curves for recombinant TNF-*α* and IL-6.

### 2.8. Western Blot Analysis of Lung Tissue

The lungs were harvested at 6 h after LPS administration. After removing extrapulmonary structures, lung tissue was frozen in liquid nitrogen immediately until homogenization. Tissue extracts were homogenized in tissue protein extraction solution (T-PER; Pierce, Rockford, Ill, USA) containing 1% proteinase inhibitor cocktail. After centrifugation, the protein concentration in the supernatant was determined by Bradford assay [12]. Each well was loaded with 100 *μ*g of protein, separated by SDS-PAGE, and electrophoretically transferred to polyvinylidene difluoride membrane. The membranes were blocked with 5% (w/v) nonfat dried milk for 1 h at room temperature to reduce nonspecific binding, washed with PBS containing 0.1% Tween-20 (PBST), then probed with antibodies including iNOS, COX-2, GAPDH, and phosphorylated and nonphosphorylated forms of Akt. After the membranes were washed again with PBST, a 1 : 10 ,000 (v/v) dilution of horseradish peroxidase-labeled IgG was added at room temperature for 1 h, and the blots were developed using ECL western blotting reagents.

### 2.9. NF*κ*B Activation

Nuclear extract from lung homogenates was measured by a nuclear extraction kit (NE-PER Nuclear and Cytoplasmic Extraction Reagents, Thermo Science) according to the manufacturer's instructions, and the protein concentration in the extract was determined using a Bradford assay dye reagent (Bio-Rad). To detect NF*κ*B activation, a NF*κ*B p65 Transcription Factor Kit (Thermo Science) was used according to the manufacturer's instructions, in which 10 *μ*g of nuclear proteins were added to the NF*κ*B binding element coated 96-well plate for 1 h, incubated with anti-p65 antibody, and followed by a secondary antibody linked to horseradish peroxidase. Plates were then developed with a chemiluminescent substrate and read in a microplate reader.

### 2.10. Statistical Analysis

Statistical analyses were performed using ANOVA followed by the Bonferroni *t* test for multigroup comparisons; *P* < 0 .05 was considered significant for all tests.

## 3. Results

### 3.1. Effects of Luteolin on LPS-Induced Histological Changes in Lung

**Figure 1:**
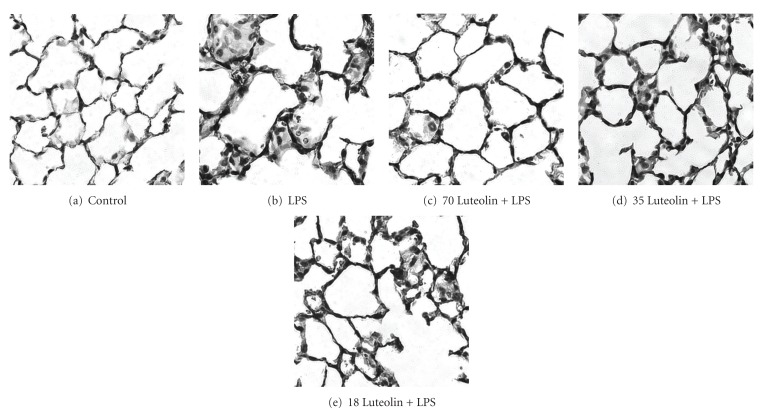
Effect of luteolin on LPS-induced histopathologic alterations in lung (400X). Histopathologic study of the lungs was performed at the end of 6 h right after LPS instillation. The lungs were removed immediately after the mice were sacrificed by thoracotomy, fixed in paraformaldehyde, embedded in paraffin, sectioned at 3 *μ*m, and stained with H&E before observation. (a) Vehicle-treated/LPS (−) group; (b) vehicle-treated/LPS (+) group; (c) 70, (d) 35, and (e) 18 *μ*mol/kg luteolin-treated/LPS (+) group.

To evaluate the effects of luteolin on ALI, we examined the histological changes occurred in lung in LPS administered mice with or without luteolin pretreatment. Under light microscope, the sham operation group expressed normal lung structures and no damage was observed (Figure 1(a)). As shown in Figure 1(b), administration of LPS for 6 h without luteolin pretreatment caused extensive morphological damages in lung which were manifested by increased in alveolar congestion, hemorrhage, leukocytes infiltration into alveolar space, alveolar septum thickness, and hyaline membrane formation (Figure 1(b)). With 30 min of luteolin pretreatment, we found that LPS-induced histopathological damages were inhibited in a concentration-dependent manner (Figures 1(c)–1(e)). These results demonstrated that luteolin exerted protective effect in LPS-induced ALI mice.

### 3.2. Effects of Luteolin on LPS-Induced Vascular Permeability and Edema in Lung

**Figure 2:**
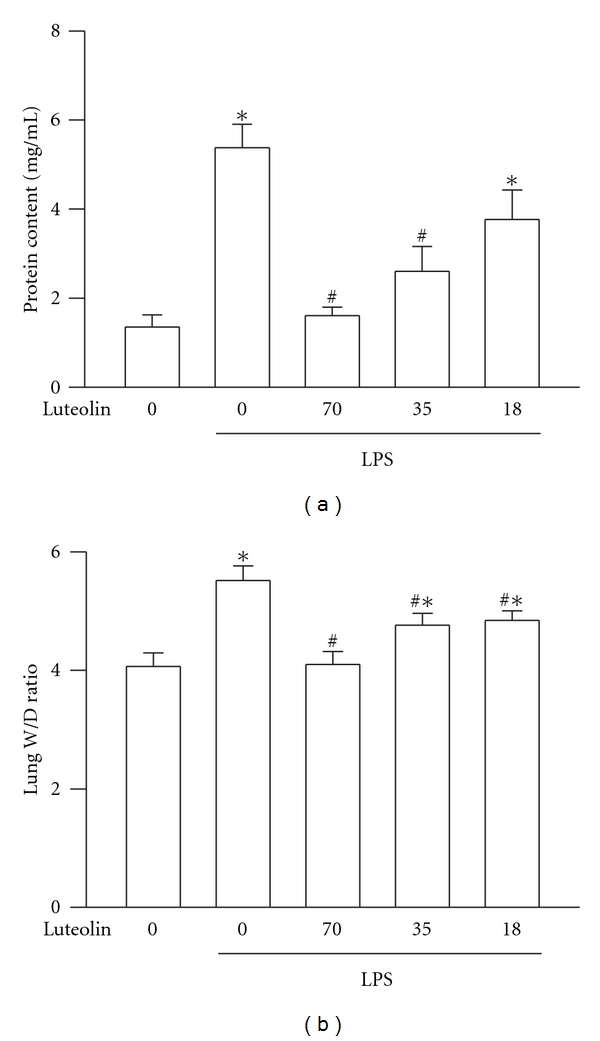
Effect of luteolin on LPS-induced PMNs and protein accumulation in BALF. (a) PBS or 18–70 *μ*mol/kg of luteolin were intraperitoneally injected for 30 min prior to intratracheal instillation of LPS (100 *μ*g/50 *μ*L saline) or saline in mice. Six hours later, the mice were anesthetized and BALF was collected for PMNs count. (b) Pulmonary permeability was determined by quantitating the protein content in cell-free BALF. (c) Pulmonary edema was determined by quantitating the wet/dry weight ratio. Values are expressed as means ± S.D. (*n* = 5 in each group). *P < 0 .05 versus control; ^#^ *P* < 0 .05 versus LPS group.

The critical feature of LPS-induced ALI is the destruction of vascular integrity, and the subsequent upregulated permeability will result in protein leakage and pulmonary edema [2]. Treatment with LPS alone was found to significantly increase protein leakage in BALF (*P* < 0 .05) and edema in pulmonary parenchyma (*P* < 0.05) in comparison with those of the sham operation group (Figure 2), while with pretreatment of luteolin at 35 and 70 *μ*mol/kg significantly reduced protein leakage and pulmonary edema (*P* < 0.05). These results indicated luteolin prevented LPS-induced pulmonary permeability and edema (Figure 2).

### 3.3. Effects of Luteolin on LPS-Induced TNF-*α* and IL-6 Production in BALF

**Figure 3:**
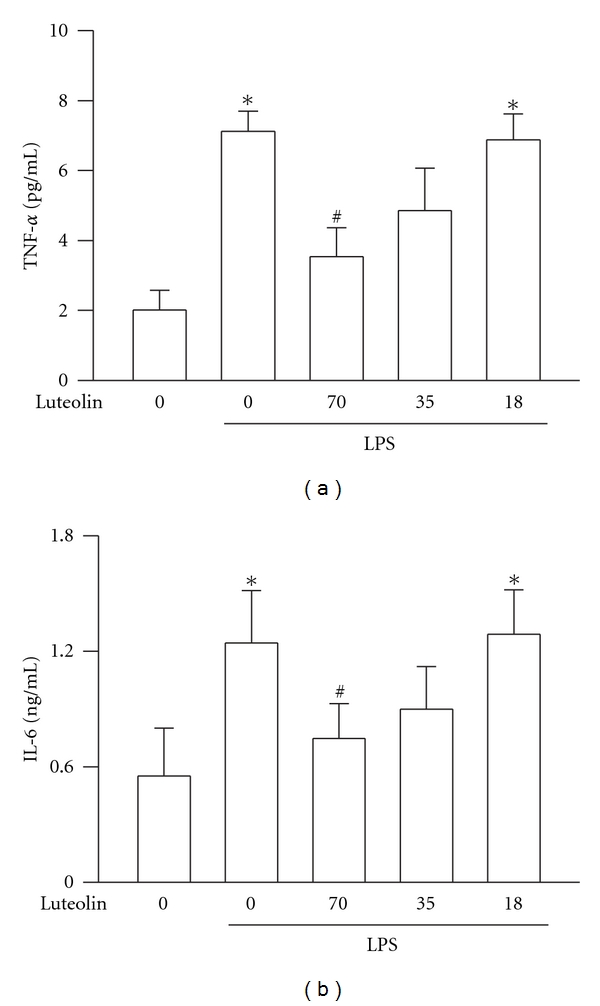
Effect of luteolin on LPS-induced TNF-*α* and IL-6 production in BALF. Values are expressed as means ± S.D. (*n* = 5 in each group). *P < 0 .05 versus control; ^#^ *P* < 0 .05 versus LPS group.

TNF-*α* and IL-6 are important mediators in recruitment of leukocytes into the lungs in LPS-induced ALI [3, 13]. The effect of luteolin on TNF-*α* and IL-6 production in the BALF was analyzed by ELISA at the end of 6 h LPS-treated period. Though LPS significantly increased the concentration of TNF-*α* and IL-6 when compared with that of the control group (*P* < 0.05), pretreatment of luteolin at 70 *μ*mol/kg significantly attenuated the production of TNF-*α* and IL-6 (*P* < 0.05, Figure 3). Furthermore, luteolin reduced LPS-induced TNF-*α* and IL-6 production in a dose-dependent manner.

### 3.4. Effects of Luteolin on LPS-Induced iNOS and COX-2 Expressions

**Figure 4:**
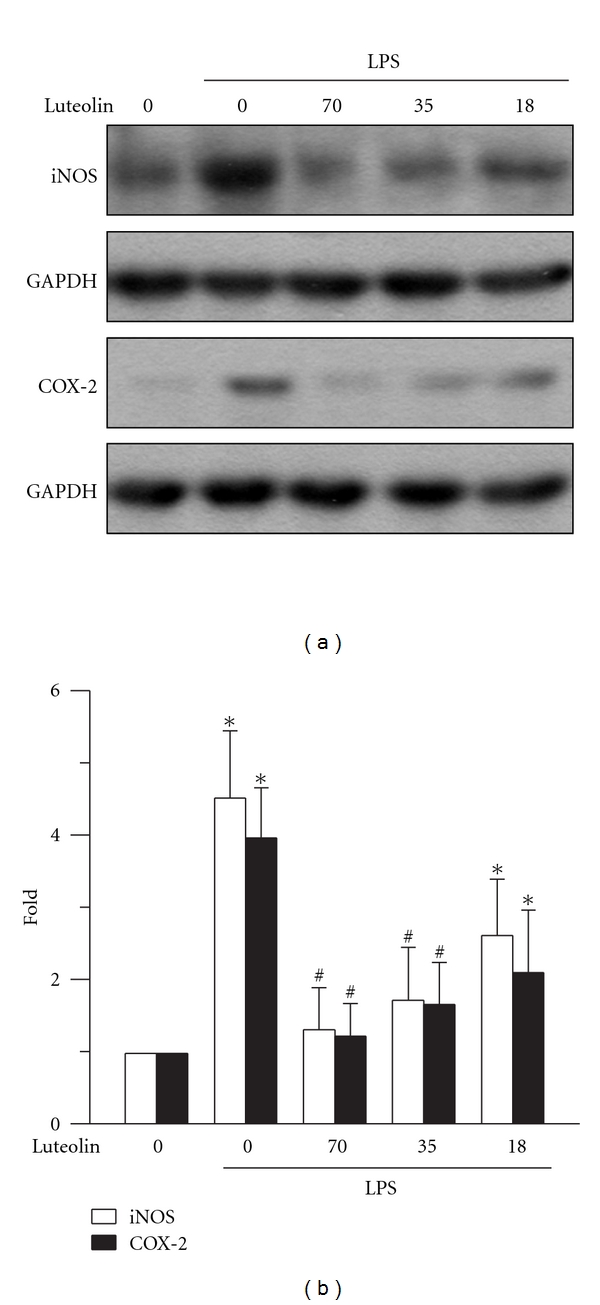
Effect of luteolin on LPS-induced iNOS and COX-2 expression in lung. Lungs were harvested from posttreated mice and whole tissue extracts were subjected to SDS-PAGE Western blot analysis using antibodies for iNOS, COX-2, and GAPDH. The ratio of immunointensity between iNOS/GAPDH and COX-2/GAPDH was calculated. The fold increases in the immunointensity is expressed as means ± S.D. (*n* = 3–5 in each group). *P < 0 .05 versus control; ^#^ *P* < 0 .05 versus LPS group.

iNOS and COX-2 play critical roles in the pathology of LPS-induced ALI [14, 15]. The effect of luteolin on iNOS and COX-2 expressions in lung tissue was analyzed using a Western blot assay. The LPS significantly increased iNOS and COX-2 expressions compared with those of the control group (*P* < 0.05). Pretreatment with luteolin reduced LPS-induced expressions of iNOS and COX-2 in a concentration-dependent manner, both 35 and 70 *μ*mol/kg significantly attenuated the expression of the two proteins (*P* < 0.05) (Figure 4).

### 3.5. Effects of Luteolin on LPS-Induced NF*κ*B Activation

**Figure 5:**
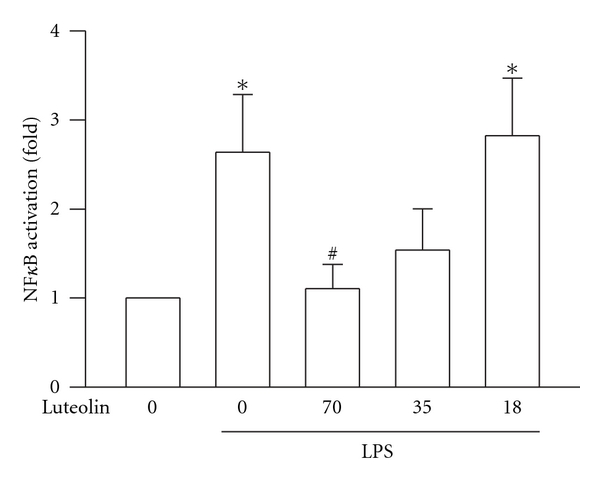
Effect of luteolin on LPS-induced NF*κ*B activation in lung. Lungs were harvested from posttreated mice, and nuclear extracts from whole lung tissues were analyzed by p65 transcription Factor assay to study the activation levels of NF*κ*B. The fold of NF*κ*B activation between the treatment and control groups was calculated. Values are expressed as means ± S.D. (*n* = 3–5 in each group). *P < 0 .05 versus control; ^#^ *P* < 0 .05 versus LPS group.

The activation of NF*κ*B, which improves transcription of most proinflammatory molecules, including TNF-*α*, IL-6, iNOS, and COX-2, has a pivotal role in ALI pathogenesis [16]. To evaluate the effects of luteolin on LPS-induced NF*κ*B activation in nuclear extracts from lung homogenates, which were analyzed using NF*κ*B p65 Transcription Factor kit. The p65/p50 heterodimer is the most abundant NF*κ*B in LPS-induced toll-like receptor signaling [17]. Because p50 lacks a transcriptional activation domain, we used p65 as the indicator for NF*κ*B activation. While the activity of NF*κ*B increased markedly after LPS administration when compared with that of the control group (*P* < 0.05), luteolin pretreatment reduced this activation in a concentration-dependent manner with an IC_50_ value of 35.1 ± 15.8 *μ*mol/kg. But a significant inhibition of NF*κ*B activation was only observed at 70 *μ*mol/kg (*P* < 0.05) (Figure 5).

### 3.6. Effects of Luteolin on LPS-Induced Akt Activation

**Figure 6:**
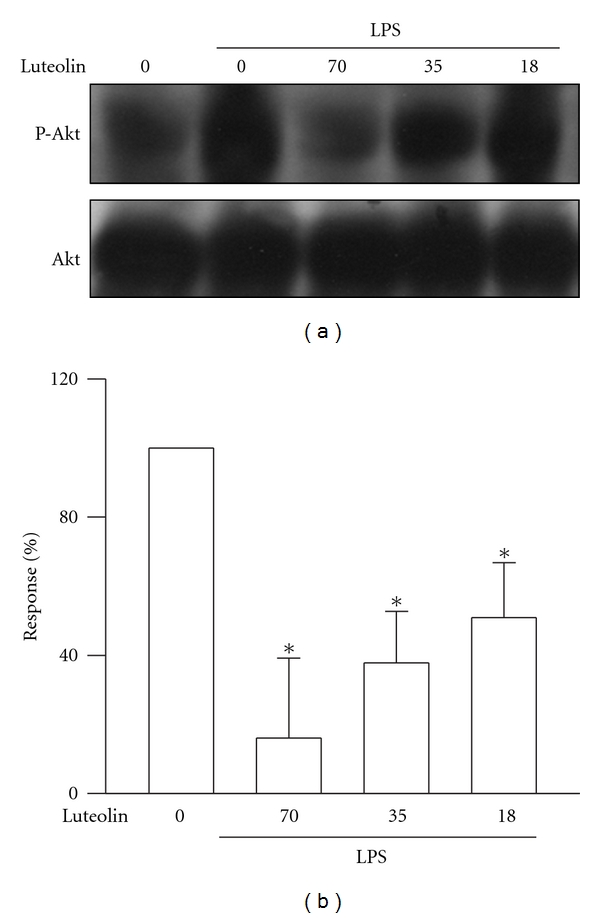
Effect of luteolin on LPS-induced Akt activation in lung. Lungs were harvested from posttreated mice and whole tissue extracts were subjected to SDS-PAGE Western blot analysis using antibodies against phosphorylated and total Akt. The ratio of immunointensity between the phosphorylation and total protein was calculated. Values are expressed as means ± S.D. (*n* = 3–5 in each group). *P < 0 .05 versus LPS group.

As an upstream factor in NF*κ*B activation, Akt participates in LPS-induced ALI [6]. Phosphorylation on Akt S473 represents its maximal activation [18]. Therefore, the effect of luteolin on Akt activation in lung tissue was assessed by phosphorylation of Akt at the site of S473 via western blot analysis. Administration of LPS on mice significantly increased Akt phosphorylation compared with that of the control group (*P* < 0.05), and luteolin reduced LPS-induced Akt phosphorylation in a concentration-dependent manner with an IC_50_ value of 30.5 ± 17.8 *μ*mol/kg (Figure 6). These results suggested that luteolin reduced the severity of LPS-induced ALI via inhibition of the PI3 K/Akt pathway.

## 4. Discussion

Experimental results obtained by this study demonstrated that luteolin contributed to a preventive effect on I.T. administration of LPS-induced inflammatory responses such as alveolar congestion, haemorrhaging, leukocytes infiltration, increase of alveolar wall thickness, protein leakage, and edema in the lungs. We found the protection mechanism of luteolin against LPS-induced ALI was via the suppression of TNF-*α* and IL-6 productions, iNOS and COX-2 expressions, and NF*κ*B and Akt activation.

ALI is a clinical syndrome induced by multiple risk factors and pathogens. Therefore, there is no ideal animal model for ALI. The symptoms of LPS-induced ALI expresses by the mouse model have close resemblance to the observed pathology in human, even if the model cannot precisely repeat all features of ALI [19]. LPS, an important virulent macromolecule, consists of three regions: lipid A, which is enclosed in the outer membrane; the core oligosaccharide, which exists as a bridge between lipid A and O-antigen; O-antigen, which is the outermost region. The toxic segment of LPS is lipid A; core oligosaccharide and O-antigen are the immunogenic but nontoxic portions. LPS is a primary trigger for innate immunity and acute proinflammatory responses. Therefore, LPS, a potent inducer of lung injury, can be employed to investigate ALI. To ensure that a real dose is delivered to the lungs of each animal, LPS was administrated directly into the lungs via trachea [20]. Exposure to LPS injures pulmonary vascular integrity, which is the most important initial cause of ALI, results in hemorrhage, protein leakage, and leukocytes infiltration in lung. These changes contribute to the development of hyaline membrane and congestion of alveolar spaces [21]. In addition, LPS increases secretion of proinflammatory cytokines, including TNF-*α*, IL-8, and IL-6, in alveolar macrophages and bronchial epithelial cells [22, 23]. These proinflammatory cytokines are crucial to leukocytes activation and recruitment into the infected site [3]. Leukocytes activation produces reactive oxygen species and protease that leads to alveolar barrier disruption, permeability escalation, and direct tissue injury [24]. Previous studies have demonstrated that pretreatment with luteolin abolished the LPS-induced accumulation of leukocytes in the air space [10, 11], and the *in vitro* study has also demonstrated that luteolin can significantly lower formyl-Met-Leu-Phe-induced neutrophils chemotaxis [11]. Oral administration of luteolin reduced bleomycin-induced total cells and neutrophils proportion in BALF [25]. At present, we also found I.P. administration of luteolin suppressed LPS-induced influx of leukocytes into alveolar space. Moreover, luteolin prevented alveolar congestion, hemorrhage, increased in alveolar septal thickness, and hyaline membrane formation caused by LPS. We also demonstrated the ability of luteolin to inhibit LPS-induced pulmonary permeability and edema. These results indicated that luteolin might have beneficial anti-inflammatory effects on animal model of LPS-induced ALI.

Proiinflammatory cytokines TNF-*α* and IL-6 not only play critical roles but also contribute to the severity of lung injury in ALI/ARDS patients [26]. The earliest and primary endogenous mediator in the inflammatory process is TNF-*α*, which primarily originates from alveolar macrophages. The binding of TNF-*α* with receptors in lung tissue leads to lysosomal leaking, directly resulting in the disruption of pneumoangiogram vascular endothelial cells and increasing their permeability. Moreover, TNF-*α* stimulates alveolar epithelial cells to generate another proinflmmatory cytokine such as IL-6 [27]. Both TNF-*α* and IL-6 induce adhesion molecule expression in vascular endothelial cells, resulting in recruitment of leukocytes into inflammatory site. Therefore, TNF-*α* and IL-6 serve as predictive markers for ALI severity. Many sequelae associated with ALI result from persistent elevation of proinflammatory cytokines in serum and BALF [28]. Previous studies have shown luteolin inhibits LPS-induced TNF-*α* and IL-6 release from alveolar macrophage [10, 29]. Studies done with mice model have shown that bleomycin stimulated production of TNF-*α* and IL-6 is reversed by luteolin [25]. Experimental data from this study demonstrated that pretreatment with luteolin downregulated expressions of TNF-*α* and IL-6 in the BALF of mice been challenged with LPS for 6 h afterwards. This result implied that luteolin confers protection to mouse ALI induced by LPS through reducing the production of TNF-*α* and IL-6.

Expression of iNOS, which generates NO, and COX-2, which generates prostaglandins and thromboxanes, contributes to the pathophysiological progression of ALI [3, 30, 31]. In mouse alveolar macrophages, luteolin pretreatment suppresses expression of iNOS and COX-2 after LPS administration [29]. An *in vivo* assay indicates that luteolin can reverse the expression of iNOS and COX-2 if pretreated by bleomycin [25]. According to our results, we concluded with fair certainty that luteolin inhibited LPS-induced increases in iNOS and COX-2 expressions in lung.

The expression of iNOS, COX-2, TNF-*α*, and IL-6 in lung are regulated by NF-*κ*B activation, which participates in the regulation of the expression of multiple immediate early genes involved in the acute inflammatory responses [32, 33]. NF-*κ*B activation is stimulated by LPS through activation of Akt [34], which is the major downstream molecule of phosphoinositide 3-kinases. Its full activation requires S473 phosphorylation in a hydrophobic motif [18]. Therefore, Akt and PI3 K participate in LPS-induced ALI [6]. Luteolin prevents Akt phosphorylation and NF-*κ*B activation in LPS-stimulated alveolar macrophages [29]. In our present study, the calculated IC_50_ values of luteolin obtained from the inhibition of both Akt phosphorylation and NF-*κ*B activation are similar. These results suggested that luteolin reduced the LPS-induced ALI by inhibiting NF-*κ*B activation probably via the PI3K/Akt pathway.

In conclusion, pretreatment with luteolin in the mouse model markedly attenuated pulmonary inflammation in ALI caused by LPS via I.T. administration. The manifestations of pulmonary inflammation are as follows: (1) pathological changes due to lung damage, such as increased in alveolar congestion, hemorrhage, leukocytes infiltration into alveolar space, alveolar septum thickness, and hyaline membrane formation; (2) elevation of lung permeability and tissue edema; (3) production of TNF-*α* and IL-6; (4) iNOS and COX-2 expressions in the lung. The molecular mechanism by which luteolin protects the lung from injury is by inhibiting NF-*κ*B activation possibly via the PI3K/Akt pathway. Experimental findings support the potential use of luteolin as a therapeutic agent for prevention of ALI associated with direct infection by Gram-negative bacteria.
